# Lower Eyelid Blepharoplasty With Volume Preservation Using the Skin Flap

**DOI:** 10.1093/asjof/ojad074

**Published:** 2023-08-07

**Authors:** Gabriele C Miotto, Orr Shauly, Ambika Menon

## Abstract

Lower blepharoplasty is one of the most commonly performed facial plastic surgeries and can be approached through many different techniques to improve perceived aging and fatigue due to prominent eyelid fat pads, deep tear troughs, loose eyelid skin, and global periorbital deflation. The technique discussed herein is a personal approach to lower eyelid rejuvenation surgery by the senior author, focusing on volume preservation with conservative resection and transposition of lower eyelid fat pads, muscle preservation, and microfat grafting. The authors discuss a series of 80 patients with case examples and a technique video.

**Level of Evidence: 5:**

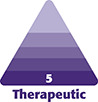

Lower blepharoplasty is one of the most commonly performed facial plastic surgeries and includes a plethora of different surgical techniques to improve lower eyelid aging and the tired look that patients perceive due to prominent eyelid fat pads, deep tear trough, loose eyelid skin, and periorbital deflation.^1,2^ Historically, lower eyelid blepharoplasty has been a reductionist procedure, with focus on skin, muscle, and fat pad resection instead of eyelid rejuvenation and beautification.^3^ However, modern eyelid surgery focuses on restoring the youthful lower eyelid shape by preserving periorbital volume.^1,2,4^ This can be accomplished through techniques that involve the transposition of fat pads, the addition of fat grafting, conservative skin excision or resurfacing, and muscle preservation.^4-7^ Conservation of tissue is intuitive for preservation and restoration of periorbital volume loss, which is one of the main components of aging. Minimizing complications and distortion of the natural anatomy is another important point in the rejuvenation of the eyelids.^1,5,8^

The technique discussed herein is a personal approach to lower eyelid rejuvenation surgery by the senior author. It focuses on volume preservation with conservative resection and transposition of lower eyelid fat pads, preservation of orbicularis oculi muscle, addition of microfat to deflated periocular compartments, and conservative skin excision.^1,2,4,5,7,8^ Lower eyelid shape improvement with superficial retinaculum suspension is another component of eyelid beautification and rejuvenation and is used in many cases to enhance aesthetic outcomes and prevent lower eyelid malposition.^9,10^

## PREOPERATIVE PLANNING

It is important to evaluate the 5 major components of the periocular region—the anterior lamella, orbital fat pads, lateral canthal shape, brow position, and periocular fat compartments. The anterior lamella includes the skin and orbicularis oculi muscle. Common signs of aging in this anatomical area include thinning of the skin, wrinkling, skin laxity, muscle laxity, festoons, and malar bags. The orbital fat pads not only become pseudoherniated with age, due to tissue laxity but also periorbital deflation, causing a visible separation of the lid cheek junction and the tired look. The lateral canthal shape and canthal tilt are always evaluated in lower eyelid surgery, since their modification and suspension can have a very positive effect on global eyelid rejuvenation and attractiveness. The eyebrows contribute to periorbital aging and indirectly affect the lateral “c-angle” and lateral canthal visibility.^11^

The understanding of the periorbital fat compartments in eyelid rejuvenation and beautification has been described by several authors, but Val Lambros’ model of facial aging really transformed the way we think about periocular volume restoration and its major influence in eyelid rejuvenation strategies.^12^

## METHODS

### Indications

A variety of patients showing a combination of aging of the anterior lamella, prominent fat pads, need for improvement of the lateral canthal shape and support, and periocular deflation have indications for the lower lid blepharoplasty with volume preservation shown here.

All surgeries were performed under general anesthesia in an American Association for Accreditation of Ambulatory Surgery Facilities–certified ambulatory surgery center. Patients provided written consent for image sharing.

The lower eyelid technique presented here (Video) consists of a combination of well-described periorbital rejuvenation techniques that aim to achieve:

1. Improvement of the lower eyelid anterior lamella aging (skin wrinkling and skin bunching) by a skin only flap (detachment of the skin from the muscle) and gentle muscle coagulation with the electrocautery for mild muscle shrinkage.
2. Conservative fat pad resection of fat pseudoherniation and transposition after release of the tear trough ligament. Visual inspection of a flat surface in the preseptal lower eyelid bulge was determined as the endpoint of fat pad resection.
3. Lateral retinaculum suspension (LRS) when needed for improvement of shape or support of the lateral canthal area.^10,13^ Neutral or negative lateral canthal tilt patients undergo LRS in 100% of the cases. This enhances the lower eyelid position aesthetically and prevents further caudal displacement of the lateral canthal angle with healing. Positive canthal tilt patients undergo LRS if presenting moderate lower eyelid laxity (lower eyelid distraction test >6 mm or delayed snap back test) to prevent eyelid malpositioning and lateral canthal rounding and gapping.
4. Microfat grafting to the deflated periocular fat compartments.^14^ All lower eyelid fat compartments are routinely inspected by visual surface analysis for volume deficiency and addressed accordingly. It not only improves the aesthetic outcomes but can also improve the anatomy of the negative vector patients and give support to the lower eyelid.
5. Muscle hitch suture to tighten the lower orbicularis to improve lower eyelid shape.^9^ This technique is used when the lower eyelid orbicularis oculi is mildly redundant after the muscle shrinkage with the electrocautery and we desire further lower eyelid support and shape enhancement.

### Anterior Lamella

This lower eyelid blepharoplasty technique is ideal for patients presenting with mild-to-moderate anterior lamella laxity and wrinkling of the lower eyelid skin, mild-to-moderate skin excess, and mild muscle laxity but no large festoons or malar bags. For the treatment of patients with severe anterior lamella laxity, an “open sky” or skin muscle flap elevation and suspension would be preferred.^15^

The senior author has been using the combination of surgical steps described in this manuscript as her preferred lower eyelid blepharoplasty approach in 60% of her patients for the last 7 years. The patient age varied from 35 to 74 and 90% of the patients were females.

It is the experience of the senior author that the use of the skin flap elevation only, leaving the orbicularis muscle down and preserved, favors minimal disruption of lymphatics of the periorbital area, potentially causing less swelling, faster recovery, and lower downtime than the traditional skin muscle elevation, while achieving optimal aesthetic results.

### Orbital Fat Pads

All 3 inferior orbital fat pads should be evaluated for resection vs transposition. Most patients not only present with a variable amount of pseudoherniation of the fat pads but also loss of volume in the periorbital fat pad compartments (suborbicularis oculi fat [SOOF], superficial orbital, and cheek fat pads). Fat transposition of the lower eyelid fat pads over the orbital rim and into the midface and SOOF is a great option for volume restoration and reshaping of the lower eyelids.^15^ Excessive fat that is not transposed requires conservative resection. The resection and transposition of the fat pads with this approach are performed through a small opening in the orbicularis oculi with exposure of the septum and teasing of the fat for removal. Transposition is also possible after the release of the orbital retaining ligament (ORL), which tears through the ligament. The fat is released from the septum, positioned into the upper midface pocket, and secured in position with a 5-0 fast gut suture.

### Lateral Canthal Support

The lateral canthus should then be addressed in many patients to improve the lower eyelid support or lateral canthal shape. The canthal area can have a neutral, negative, or positive canthal tilt (when comparing the angle and position of the lateral-to-medial canthus). Depending on the amount of lower eyelid laxity, and the aesthetic desire for lateral canthal shape change (eg, from negative to positive tilt), a retinaculum suspension canthopexy/canthoplasty is our intraoperatively canthal support technique of choice. Patients with neutral or negative canthal tilt usually benefit from improvements in the lateral canthal shape. In our evaluation, these patients underwent LRS in 100% of the cases. Patients with a positive canthal tilt already have a favorable anatomy and shape of the lateral canthus. In our evaluation, these types of patients were elected to have LRS only if they present moderate lower eyelid laxity (lower eyelid distraction test of >6 mm or delayed snap back test).

### Fat Grafting

Facial deflation is one of the main components of periocular aging.^16,17^ The combination of loss of volume of the periorbital fat compartments, thinning of the tissues and pseudoherniation of the fat pads demarcate the lid–cheek junction, creating the visible “dark circle.” The addition of conservative amounts of microfat at the level of the lid–cheek junction and into periorbital fat compartments has a great positive impact in rejuvenation the lower eyelid smoothing the transition of the eyelids into the midface, especially if the patient has major midface deficiency and a negative vector.

## OPERATIVE TECHNIQUE

### Step 1: Creating Skin Flap

The lower eyelid is injected with a solution of normal saline + lidocaine + marcaine + tranexamic acid + epinephrine, and vasoconstriction is achieved before incision. A subciliary incision is made using a 15-blade at the lateral lower eyelid. Then, using straight Iris scissors, the subciliary incision is completed by detaching the skin from the pretarsal orbicularis from lateral to medial. A 6-0 Prolene suture (Ethicon; Raritan, NJ) is placed into the tarsal border of the lower eyelid to retract the lower eyelid upwards and facilitate the dissection of the skin flap.

It is very important to detach the skin from the underlying muscle all the way down to the level of the tear trough and orbitomalar ligament for both better skin redraping and further approaching the fat pads through the muscle windows. During this dissection, it is important to not leave any dermis behind; otherwise, a dermal cyst can form during healing under the skin, which will need further surgical removal.

### Step 2: Fat Pad Manipulation

Using the electrocautery on a low setting (12/12 blend), the cutmode of the electrocautery creates a small hole in the orbicularis, starting right over the lateral fat pad. With retropulsion of the globe, the septum and the area with the bulging fat become clear. Curved Iris scissors can be used next to open the septum with a clean incision, allowing the fat to be teased out easily. The fat can be extracted with the use of a q-tip or delicate forceps, and should be trimmed conservatively using the electrocautery in the coagulation mode. The lateral fat pad is the most thoroughly trimmed of all of the fat pads. When also doing a canthopexy, the lateral fat tends to bulge further, so effective trimming of the fat is important for a good flat contour at the lateral lower eyelid area.

### Step 3: Ligament Release and Fat Transposition

A second window into the orbicularis oculi is created over the bulging medial fat pad. The tear trough ligament at the orbital rim is identified and released using the electrocautery, extending laterally with the release of the ORL. A Joseph elevator is used to further release the tear trough ligament into the upper midface in a preperiosteal plane. A desmarres retractor should be used to retract the muscle and expose the dissection area. The midface dissection is tailored to different needs, but usually stops with the exposure of the origin of the levator labii superioris muscle in the maxilla. This dissection creates a midface pocket for fat transposition. Then, the septum over the nasal and central fat pads is opened to trim the excess bulging fat and create a flap with the central fat pad to be transposed. The fat removed can also be utilized for free grafting. The transposed fat fills in the depression of the tear trough and the ORL, allowing for volumization and improvement of the lid–cheek junction. The tip of the fat flap is tied with 5-0 fast gut suture, and the suture acts as a guide into the midface pocket. The needle exits from inside out through the midface skin and is anchored to the skin with steri-strip for healing.

### Step 4: Muscle Contouring

After the fat removal and fat transposition, the contours of the lower eyelid are evaluated. Muscle laxity is evaluated and often there is visible mild muscle laxity. Using the coagulation mode, the orbicularis surface is coagulated in a few areas at 12 W, which gently retracts the muscle. This is a very beneficial maneuver for patients with bulging pretarsal orbicularis. One should avoid excessive muscle coagulation to prevent anterior lamella retraction.

### Step 5: Canthopexy

Next, we perform a LRS canthopexy. The superficial retinaculum is identified by creating a muscle tunnel from the upper eyelid to the lower eyelid, at the lateral orbital rim and visualizing the superficial part of the tendon. There is a gliding plane of dissection around the superficial retinaculum, and 1 can approach it from the upper eyelid or the lateral lower eyelid incision. Using a 4-0 polydioxanone to suture, the retinaculum is looped and then anchored into the lateral orbital rim periosteum, at the level of the pupil for the majority of the patients. This anchoring position can be changed depending on the need for shape change and eyelid support.

If additional lower eyelid and muscle support is needed, a muscle suspension suture, or modified muscle hitch suture can be placed. A 5-0 Vicryl suture (Ethicon) is passed craniocaudally through the periosteum of the lateral orbital rim, coming out superficially at the lateral canthal area, exiting through the lateral lower eyelid orbicularis muscle, then returning through the muscle at the same level of the exit and diving deep back toward the lateral orbital rim where the suture started. Then, the suture is tied, giving extra support and lifting the lateral corner of the lower eyelid.

### Step 6: Skin Resection

The skin is then redraped over the lower eyelid, and the excess is evaluated and trimmed conservatively using straight iris scissors, making sure there are no major gaps between the incision and the skin flap. There is usually more removal laterally than medially.

### Step 7: Closure

The lower eyelid incision is closed using a few interrupted 6-0 fast gut sutures throughout the extension of the incision. One of the most helpful and effective ways of preventing chemosis in the author’s experience, besides gentle tissue manipulation in surgery, is the placement of a temporary tarsorrhaphy suture at the end of the case. A temporary tarsorrhaphy suture is placed as a horizontal mattress suture from the gray line of the upper eyelid, through the gray line of the lower eyelid, approximating the eyelid together at the most lateral aspect of the eyelid fissure. This suture is placed both to prevent chemosis and avoid extra eyelid dryness in the first 4 to 5 days after surgery. Steri-strips are placed over the incisions and in the dissected areas to help decrease swelling.

### Step 8: Fat Grafting

Using the microfat harvesting technique described by Tonnard et al, fat is manually harvested from the donor site (abdomen, flanks, and thighs) using a 10 cc syringe attached to a 2.4 mm cannula with 1 mm holes.^18^ The donor area is infiltrated with a solution of NS + lidocaine + epinephrine before harvesting. Once harvested, the fat is strained gently to remove excess fluid and oil and placed into 1 cc syringes. Then, using blunt cannulas of 0.7, 0.9, and 1.2 mm (Tulip set reference), the fat is injected smoothly into the periorbital fat compartments. An 18G needle is used to create the skin entrance point for the cannula. The fat is injected with a fanning technique, with low pressure and low volume into all compartments that need revolumization, starting with the deep malar fat pad and SOOF, and then into superficial orbital and midface compartments. Low pressure and gentle handling of the fat avoid bolus injection of fat to reduce the risk of fat necrosis and oil cysts.

### Postoperative Protocol

Once the surgery is finished and the tarsorrhaphy is in place, the periorbital area (midface, malar area) is taped using skin-colored Steri-strips for gentle compression as the tissues heal. Taping has been a wonderful add-on to the recovery of our patients, as it seems to help significantly with bruising and swelling control. Icing of the eyelids is recommended for 15 min every hour when the patient is awake, at least for the first 5 days after surgery. Sleeping with the head gently elevated (2 pillows) is also recommended for 5 to 7 days. On the first postoperative day, all patients are seen on video as a televisit. Then, on Days 4 and 5, patients come to the office for tarsorrhaphy suture removal and tape removal. A low-salt and low-sugar diet, along with good hydration and good protein intake, is recommended starting before surgery and continuing for 4 weeks, and hopefully for a lifetime, as a recommendation for a healthy lifestyle.

### Complications

In the senior author's experience, complications have been infrequent but can happen. All steps aim toward precise tissue resection and repositioning to prevent the feared lower eyelid surgery complications. In this series, the following complication rates were observed: lower eyelid retraction and malpositioning with scleral show (1/80), infection (1/80), chemosis (1/80), lower eyelid retraction (0/80), dry-eye syndrome (0/80), dermal cyst (1/80), oil cyst (1/80), fat pad under resection (8/80), and excessive fat grafting absorption (8/80). Our complication rates are shown in Table.

**Table.** Complications Across a Series of 80 Total Patients in the Senior Author's Practice

Complication	Rate (%)
Scleral show	1.125
Dermal cyst	1.125
Oil cyst	1.125
Infection	1.125
Chemosis	1.125
Fat pad under resection	10
Dry-eye syndrome	0
Lower eyelid retraction	0
Excessive fat grafting absorption	10

From the complications listed above, the chemosis was treated conservatively with eye steroid drops for 5 days and night-eye lubricant and patching of the affected eye for 2 weeks. The oil cyst was drained transconjunctively in the operating room under sedation. The dermal cyst was removed in the office under local anesthesia through the subciliary approach. Regarding the fat pad under resection, 3 patients elected to undergo further resection of the residual fat pad in the operating room under sedation. The scleral show was mild, and the patient elected not to have further intervention. Fat grafting absorption was treated in 3 patients with the addition of volume to the midface and SOOF compartments using a combination dermal hyaluronic acid filler and Renuva adipose matrix (MTF Biologics; Edison, NJ) 6 months after surgery.

## CASES

### Case 1

**Figure 1.**
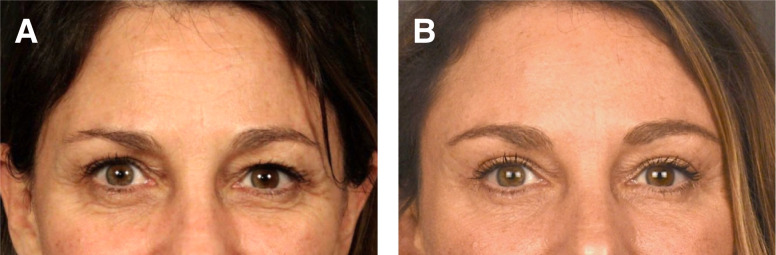
(A) Preoperative photograph of a 51-year-old female and (B) 6 months postoperative skin flap and muscle tightening lower blepharoplasty, and skin only upper blepharoplasty, retinaculum suspension of the lateral canthus, lateral endoscopic brow lift, and microfat grafting of the deep malar, suborbicularis oculi fat (SOOF), superficial cheek, upper eyelids, and temples.

This is a 51-year-old female 6 months before and after skin only upper and lower blepharoplasty, superficial retinaculum suspension, lateral endoscopic brow lift, and microfat grafting to the deep malar, SOOF, superficial cheek, upper eyelids, and temples (Figure 1).

### Case 2

**Figure 2.**
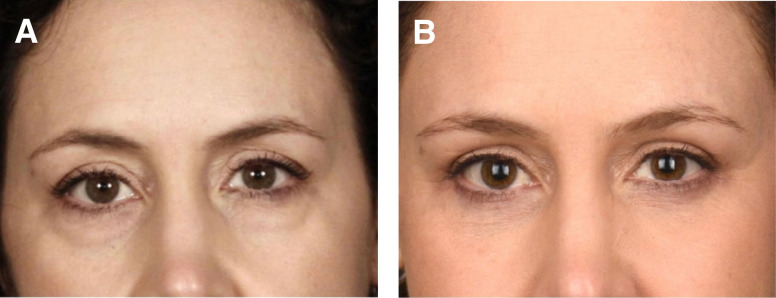
(A) Preoperative photograph of a 59-year-old female and (B) 6 months postoperative skin flap and muscle tightening lower blepharoplasty and upper blepharoplasty, lateral endoscopic brow lift, and microfat grafting of the deep malar, superficial retinaculum suspension, and upper eyelids.

This is a 46-year-old female 6 months before and after skin only upper and lower blepharoplasty, lateral endoscopic brow lift, microfat grafting of the deep malar, and superficial retinaculum suspension (Figure 2).

### Case 3

**Figure 3.**
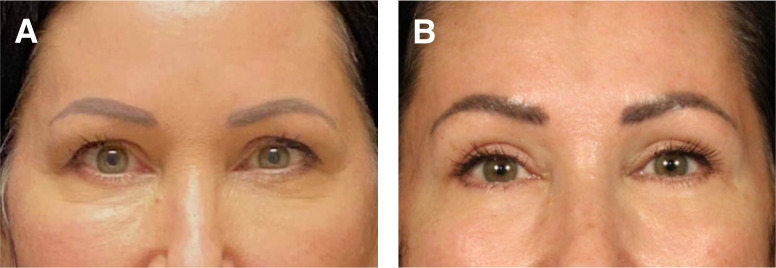
(A) Preoperative photograph of a 53-year-old female and (B) 10 months postoperative skin flap and muscle tightening lower blepharoplasty, skin only upper blepharoplasty, removal of central and lateral fat pads, lateral endoscopic brow lift, and microfat grafting of the deep malar, suborbicularis oculi fat (SOOF), superficial cheeks, and temples.

This is a 53-year-old female 10 months after postoperative skin flap and muscle tightening lower blepharoplasty, skin only upper blepharoplasty, removal of central and lateral fat pads, lateral endoscopic brow lift, and microfat grafting of the deep malar, SOOF, superficial cheeks, and temples (Figure 3).

### Case 4

**Figure 4.**
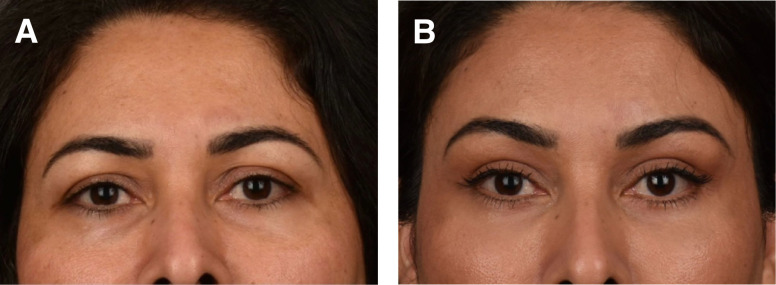
(A) Preoperative photograph of a 43-year-old female and (B) 6 months after skin only upper and lower blepharoplasty, lateral brow lift, superficial retinaculum suspension, fat pad removal and transposition of central lower eyelid fat pad, and microfat grafting of the deep malar, suborbicularis oculi fat (SOOF), upper eyelid, temples, and superficial cheek.

This is a 43-year-old female 6 months before and after skin only upper and lower blepharoplasty, lateral brow lift, superficial retinaculum suspension, fat pad removal and transposition of central lower eyelid fat pad, and microfat grafting of the deep malar, SOOF, upper eyelid, temples, and superficial cheek (Figure 4). This patient is featured in the attached surgical Video.

## Supplementary Material

ojad074_Supplementary_Data
